# Clinic of Dr. Gregory at St. Louis Sisters’ Hospital

**Published:** 1872-06

**Authors:** 


*Clinic of Dr. E. H. Gregory, at St. Louis Sisters' Hospital.*

"*Prolapsus Ani.*"—About the first of March, 1871, Johnny, age seven years, suffering from prolapsus of the rectum, was brought to the Sisters' Hospital and placed in the ward under the charge of Prof. Gregory. This was one of those exceptionally bad cases, fortunately not frequently met with. The intestine protruded, upon the least straining and after each evacuation of the bowels, at least six or seven inches. When brought to the hospital he (the child) presented a remarkably healthy appearance, and, according to the statements of the mother, he had always been a very healthy child. Upon questioning her as to the cause, it was ascertained that she had one of those little *closet chairs*, upon which she was accustomed to set the child when he desired to go to stool, placing his toys before him on the little table—which, by the way, is to mothers the attractive feature of the chair—she allowed him to sit there and strain until he became tired; thus, it was not difficult to determine the cause of this horrible condition. The treatment was at first palliative, and consisted in keeping the bowels slightly relaxed and forcing him to evacuate his bowels in the horizontal position. Having derived no benefit from this treatment, it was decided to apply the actual cautery to the margin of the anus in three or four places, great pains being taken to avoid the mucous membrane of the intestine; the bowel being with much difficulty prevented from prolapsing. This procedure was not in the least beneficial. In the meantime, while the wounds were healing, a stringent enemata, consisting of tinct. ferri muriat. dr. j, aquæ destil. oz. j, divided into five injections, were given two or three times a day: no benefit being derived from these, they were discontinued after a thorough trial. Again, after the lapse of several weeks it was decided to operate: this time by ligaturing portions of the mucous membrane over the protruded surface. This was done by seizing, here and there, portions of the membrane with the forceps, raising it from the muscular coat and applying a ligature. By this the prolapse was shortened one or two inches; the bowel was then returned and the separation of the ligatures awaited. It was two or three weeks before the intestine again protruded, and then not so much of it as formerly. After some time another operation was deemed necessary, and, being encouraged by the result of the last, it was thought best to again endeavor to shorten the protruding bowel; this time by passing three or four sutures in a horizontal direction under the mucous membrane, enclosing about an inch of the membrane in each loop. After this operation the prolapsus was less frequent, and protruding portion much shortened. Still it required two more operations to make the cure perfect. In both of these the cautery was again brought into requisition. On both occasions, the irons, heated to a white heat, were drawn slowly over the prolapsed intestine four or five times, in a direction corresponding to the axis of the bowel, thus making linear eschars through the mucous membrane. After the last application of the cautery the cure was complete, and the boy left the hospital about the first of August, to all appearances permanently cured. Since then he has been heard from several times; and up to the time when last heard from, several weeks since, there had been no return of the disease.

Upon consulting the different authorities on the subject, I find that all, at first, recommend palliative measures, and without exception they agree that all cases, except the very bad ones of long standing, may be cured without surgical interference. This consists in improving the general health—if that be impaired—removing the exciting cause, and requiring the stools to be voided either in the horizontal position or standing. Van Buren, in his lectures upon diseases of the rectum, cites a case cured by a negro nurse, who forced the child to have her evacuations standing. The disease returned again after a severe attack of dysentery, and, the above plan failing, the child was brought to him, and he required the evacuations to be had in the horizontal position: the result was a permanent cure.

When this condition is dependent upon atony, atrophy, or fatty degeneration of the external sphincter, electricity is recommended as being beneficial. Cases are occasionally met in which the prolapsus is difficult to return; great care should then be exercised in order not to bruise the bowel, as Cruveilhier mentions a case of this kind which required much force in its reduction. The patient, next day, showed serious symptoms, suppurative phlebitis having set in. Five days later he died, and the post mortem revealed the veins around the rectum inflamed, and also small abscesses in the liver.

In those cases requiring operations, Dupuytren was in the habit of removing three or four elliptical folds from around the margin of the anus, involving a small portion of the mucous membrane within and the skin without, and then drawing the edges together with sutures. Gross says he assisted in removing two large triangular pieces, one from each side of the anus, and then drawing the cut surfaces together with sutures. This operation was not successful, the condition of the patient remaining unaltered. The same authority speaks of having operated successfully by raising portions of the mucous membrane and applying the ligatures. He says this operation may have, in some cases to be repeated, but that it always succeeds eventually.

Valentine Mott modified Dupuytren's operation by removing several pieces of mucous membrane from the surface of the prolapse, and drawing the edges together with sutures, at the same time removing the elliptical folds from the verge of the anus. Van Buren, after cutting away pieces of mucous membrane, as recommended by V. Mott, applies the button cautery, heated to a dull red heat, to the margin of the anus; he also recommends the cautery to be applied to the surface of the protruded intestine. Sir. Benj. Brodie advises the amputation of the prolapse in those cases not benefited by the ordinary operations. The actual cautery seems at present to have almost entirely excluded the knife and ligature; and it is thought to have been the principal agent in the cure of the above case.—*St. Louis Medical and Surgical Journal.*

